# *Grx2* Regulates Skeletal Muscle Mitochondrial Structure and Autophagy

**DOI:** 10.3389/fphys.2021.604210

**Published:** 2021-03-05

**Authors:** Ava Liaghati, Chantal A. Pileggi, Gaganvir Parmar, David A. Patten, Nina Hadzimustafic, Alexanne Cuillerier, Keir J. Menzies, Yan Burelle, Mary-Ellen Harper

**Affiliations:** ^1^Department of Biochemistry, Microbiology and Immunology, Faculty of Medicine, University of Ottawa, Ottawa, ON, Canada; ^2^Ottawa Institute of Systems Biology, University of Ottawa, Ottawa, ON, Canada,; ^3^Faculty of Health Science, Interdisciplinary School of Health Sciences, University of Ottawa, Ottawa, ON, Canada

**Keywords:** mitochondria, glutathione, mitochondrial dynamics, disulfide relay system, glutaredoxin 2, autophagy

## Abstract

Glutathione is an important antioxidant that regulates cellular redox status and is disordered in many disease states. Glutaredoxin 2 (*Grx2*) is a glutathione-dependent oxidoreductase that plays a pivotal role in redox control by catalyzing reversible protein deglutathionylation. As oxidized glutathione (GSSG) can stimulate mitochondrial fusion, we hypothesized that *Grx2* may contribute to the maintenance of mitochondrial dynamics and ultrastructure. Here, we demonstrate that *Grx2* deletion results in decreased GSH:GSSG, with a marked increase of GSSG in primary muscle cells isolated from C57BL/6 *Grx2^−/−^* mice. The altered glutathione redox was accompanied by increased mitochondrial length, consistent with a more fused mitochondrial reticulum. Electron microscopy of *Grx2^−/−^* skeletal muscle fibers revealed decreased mitochondrial surface area, profoundly disordered ultrastructure, and the appearance of multi-lamellar structures. Immunoblot analysis revealed that autophagic flux was augmented in *Grx2^−/−^* muscle as demonstrated by an increase in the ratio of LC3II/I expression. These molecular changes resulted in impaired complex I respiration and complex IV activity, a smaller diameter of *tibialis anterior* muscle, and decreased body weight in *Grx2* deficient mice. Together, these are the first results to show that *Grx2* regulates skeletal muscle mitochondrial structure, and autophagy.

## Introduction

Disordered mitochondrial glutathione reduction-oxidation reactions (redox) are associated with many disease states, such as type 2 diabetes, aging-associated neurodegenerative diseases, and rare genetic mitochondrial diseases (McMurray et al., 2016; Calabrese et al., 2017; Stockwell et al., 2017; Hoffmann and Griffiths, 2018; Kanaan et al., 2018). As a major non-protein antioxidant in cells, glutathione buffers elevations in hydrogen peroxide (H_2_O_2_), a form of reactive oxygen species (ROS) that contributes directly to the pathophysiology of these diseases (Houstis et al., 2006). Cellular ROS production occurs predominantly within the mitochondria of most cell types, making mitochondria the cellular hubs of redox reactions (Murphy, 2009; Diebold and Chandel, 2016; Murphy and Hartley, 2018; Yan et al., 2020). As such, mitochondria employ a variety of mechanisms to facilitate the detoxification of these potentially harmful reactive species. Protein glutathionylation is a rapid and reversible post-translational modification, which functions to protect vulnerable sulfhydryl groups from irreversible oxidation/damage in response to fluctuations in the redox environment (Nikolaienko et al., 2018; Figlia et al., 2020; Mailloux, 2020). In mammalian cells, enzymatic deglutathionylation is catalyzed by glutaredoxins (glutathione dependent reductase and oxidase, GRX; Holmgren, 1976; Beer et al., 2004). *Grx2* is the primary mitochondria-specific Grx isoform whose function varies in response to fluctuations in the reduced glutathione (GSH); oxidized glutathione (GSSG) ratio; a high GSH:GSSG ratio promotes protein deglutathionylation, and a low GSH:GSSG ratio promotes *Grx2* glutathionylation of target proteins (Beer et al., 2004; Hurd et al., 2008). Glutathionylation reactions can also alter protein activity thereby rendering the targets active or inactive (Adachi et al., 2004; Pimentel et al., 2006), and are particularly abundant in mitochondrial proteins rich in cysteine thiols (Mailloux, 2020).

Mitochondria are dynamic interconnected reticular structures that are continuously undergoing fusion and fission to facilitate metabolic demands and mitochondrial DNA exchange (Chan, 2020; Giacomello et al., 2020; Sabouny and Shutt, 2020; Yu et al., 2020). Fusion is dependent on the activation of large GTPase proteins, mitofusins (MFN)-1 and -2 to facilitate fusion of opposing outer membranes (Santel and Fuller, 2001; Rojo et al., 2002; Chen et al., 2003), and the optic atrophy-1 (OPA1) protein for fusion of the inner mitochondrial membranes (Frezza et al., 2006; Song et al., 2009). Mitochondrial fission requires the recruitment of dynamin-related protein-1 (DRP1), which severs mitochondrial membranes, fragmenting the mitochondrial network (Otsuga et al., 1998). Fission is necessary for mitochondrial quality control to prime damaged mitochondrial fragments for removal *via* mitophagy (Ashrafi and Schwarz, 2013), whereas fusion can occur as an acute stress-response to protect mitochondria from degradation (Tondera et al., 2009; Gomes et al., 2011). Moreover, it was recently demonstrated that the oligomerization of MFN1 and MFN2 into higher-order complexes is partly controlled by glutathione redox (Mattie et al., 2018). In support of a role for glutathione redox in mitochondrial ultrastructure, Shutt et al. (2012) showed that depletion of GSH, and increased levels of cellular GSSG stimulate mitochondrial hyperfusion. This was later demonstrated to be due in part to a redox-regulated cysteine thiol on MFN2 (Thaher et al., 2018). Importantly, *Grx2* is particularly abundant in the mitochondrial intermembrane space (IMS), and plays a central role in regulating the redox state of the IMS import and assembly protein 40 (Mia40; Kojer et al., 2015), suggesting that it may also play a key role in MFN1/2 oligomerization. Moreover, glutathione deficiency has also been implicated in the regulation of autophagy (Filomeni et al., 2010). In retinal cells, depletion of GSH promotes LC3 autophagic flux and the appearance of autophagic vacuoles (Sun et al., 2018), whereas the addition of glutathione mitigates trehalose-induced autophagy (Underwood et al., 2010).

Within skeletal muscle, transient increases in ROS commonly occur in response to fiber contractions, and can result in the glutathionylation of complex I subunits (Gill et al., 2018), contractile proteins, and tricarboxylic acid cycle (TCA) enzymes (Kramer et al., 2018). Moreover, our lab has previously demonstrated that *Grx2* plays a key role in skeletal muscle uncoupling protein 3 (UCP3)-dependent leak (Mailloux et al., 2013), and cardiac bioenergetics function and mitochondrial dynamics in mice and humans (Kanaan et al., 2018). However, the role of *Grx2* on skeletal muscle mitochondrial ultrastructure and bioenergetic function remains unclear. Therefore, the present study aimed to examine the role of *Grx2* on mitochondrial ultrastructure in skeletal muscle. Based on our previous observation of decreased glutathione redox in skeletal muscle mitochondria of *Grx2^−/−^* mice (Mailloux et al., 2013), we hypothesized that *Grx2* regulates mitochondrial structure, turnover, and function in skeletal muscle.

## Materials and Methods

### Animals

All experiments involving mice were conducted according to the guidelines and principles of the Canadian Council of Animal Care and approved by the Animal Care Committee of the University of Ottawa. Studies were conducted on male C57BL/6 wild-type (WT) and *Grx2^−/−^* (C57BL/6 background) whole body knock-out mice aged 4–6weeks. All mice were genotyped prior to experimentation to confirm knock-out of *Grx2*. Mice were housed under standard conditions with controlled temperature, humidity, and 06:00–18:00 light cycles. Mice were supplied with *ad libitum* access to a standard chow diet (44.2% carbohydrate, 6.2% fat, and 18.6% crude protein, 2018 Teklad Global Rodent Diet, Envigo). To evaluate autophagy, mice were injected intraperitoneally with colchicine (0.4mg/kg/day) or an equal volume of saline every 24h for 2days prior to sacrifice (Vainshtein et al., 2015).

### Body Composition Analyses

Fat and lean masses were measured by a non-invasive nuclear magnetic resonance imaging whole-body composition analyzer (EchoMRI-700™; Echo Medical Systems).

### Indirect Calorimetry

Mouse O_2_ consumption, CO_2_ production, respiratory exchange ratio (RER), spontaneous and wheel-running activity as well as food intake were measured in a 12 chamber Comprehensive Lab Animal Monitoring System (CLAMS) instrument (Columbus Instruments) at thermoneutrality (28°C). Mice were individually housed in chambers and acclimated for 24–48h, before collection of data from a 24h period. Mice were provided throughout with *ad libitum* access to standard chow (as above). Volitional running activity was calculated as the total number of complete wheel rotations; spontaneous activity was assessed by beam breaks (*x* and *z*) in the chambers.

### Tibialis Anterior Muscle Fiber Diameter

Dissected *tibialis anterior* muscles were embedded in optimal cutting temperature (OCT, Tissue-Tek) compound and frozen in isopentane cooled in liquid nitrogen. Muscle was cut perpendicular to the muscle fiber axis and sectioned (8μm) at −18°C using a cryotome (Leica Biosystems). Hematoxylin and eosin (H&E) staining was performed according to a standard protocol. Muscle fiber diameter was determined using image analysis using Image J software by determining horizontal muscle fiber diameter length.

### Primary Myoblast Isolation

Primary myoblasts from WT and *Grx2^−/−^* mice were isolated from the *quadriceps*, *tibialis anterior*, *soleus*, and *gastrocnemius* muscles. The pooled muscle groups were minced and treated with collagenase/dispase (C/D; Collagenase: 0.1U/ml, Dispase: 0.8U/ml, Sigma Aldrich, 11097113001) solution to liberate myoblasts. The muscles were left in C/D solution for two 15-min incubation periods at 37°C in 5% CO_2_. After each 15-min incubation period, the suspension was manually triturated 20–25 times using a 10ml serological pipette. Primary cell enrichment was achieved by employing the differential adhesion process to remove fibroblast populations (Pasut et al., 2012). Myoblasts were cultured in Dulbecco’s modified Eagle medium (DMEM) containing 20% bovine growth serum (BGS), 1% Antibiotic-Antimycotic (AA), 30ng/μl basic fibroblast growth factor (bFGF; 13256-029, Life Technologies), and 5μg/ml gentamycin sulfate.

### GSH and GSSG Determinations Using High-Performance Liquid Chromatography (HPLC)

Concentrations of GSH and GSSG in myoblasts and *tibialis anterior* muscle were determined by high-performance liquid chromatography (HPLC) using an Agilent 1100 Series instrument, equipped with a Pursuit 5 C_18_ column with a flow rate set to 1ml/min with filtered mobile phase [10% methanol, HPLC plus (Sigma, 646377), 90% ddH_2_O, 0.09% trifluoroacetic acid (Sigma, 302031)]. Serum deprived myoblasts were trypsinized and washed twice in ice-cold 1X phosphate-buffered saline (PBS) solution and counted using a Cell Countess (Thermo Fisher Scientific). Myoblasts were resuspended in 1:1 buffer [1% (v/v) trifluoroacetic acid (Sigma, 302031) and 1% (w/v) meta-phosphoric acid (Sigma, 239275) solution in mobile phase and homogenization buffer (for 25ml, 250mM sucrose, 10mM TRIS, 3mM EDTA dissolved in mobile phase, pH of 7.4) final pH of 1:1 buffer <1.0] and incubated on ice for 20min. For determinations of GSH and GSSG in muscle, frozen *tibialis anterior* samples were weighed and homogenized in 1:1 buffer with a Teflon pestle drill. The myoblast and homogenized muscle samples were then centrifuged at 14,000g for 20min at 4°C. After centrifugation, the supernatant was collected for analyses. GSH and GSSG were detected using the Agilent UV-visible wavelength detector at 215nm. Retention times were confirmed using standards, which were prepared using 0.1, 0.01, and 0.001mM of GSH (Sigma, G4251) and GSSG (Sigma, G4501) dissolved in 1:1 buffer. Absolute amounts of GSH and GSSG were determined by integrating the area under the respective peaks using a chromatogram, and values were calculated from standard curves.

### Immunofluorescence of Primary Myoblasts Mitochondrial Length

Upon 85–90% confluency, cells were trypsinized and plated for imaging. Myoblasts were placed in starvation medium (SM) for an incubation period of 14–16h prior to cell fixation; starvation was used to synchronize cell cycle and reach a quiescent state (G_0_ state). The SM consisted of DMEM supplemented with 0.1% Bovine Serum Albumin (BSA) and 1% AA. After the starvation period, cells were fixed with 4% paraformaldehyde for 15min. The cells were permeabilized and blocked using a blocking buffer containing 0.1% Triton X-100 and 1% BSA, which also included the primary antibody, TOM20 (Santa Cruz Biotechnology, sc-11415; 1:100 dilution). Oregon green 488 goat anti-rabbit (Life Technologies, O-6381; 1:100 dilution) was diluted in 1x PBS containing Hoechst counter-stain in 1:1 PBS:Glycerol mounting media. Images were obtained using a Zeiss Axiolmager M2 microscope with a Plan-Apochromat 63X/1.4 oil objective. Fifty mitochondria lengths were quantified using ImageJ in five independent experiments for each genotype. The Mitochondrial Network Analysis (MiNA) plugin was used for the semi-automated analysis of mitochondrial network complexity to determine the average number of branches per mitochondrial network (Valente et al., 2017).

### Protein Extraction

20–30mg of frozen *tibialis anterior* muscle was homogenized using a bead mill homogenizer (Fisherbrand Bead Mill 24 Homogenizer) in ice-cold modified RIPA buffer [20mM Tris-HCl (pH 7.5), 150mM NaCl, 1mM Na_2_EDTA, 1mM EGTA, 1% NP-40, 1% Na deoxycholate, 2.5mM Na pyrophosphate, 1mM *β*-glycerolphosphate, 1mM Na_3_VO4] supplemented with a protease and phosphatase inhibitor cocktail (Sigma Aldrich; P8340). Homogenates were centrifuged at 14,000g for 10min at 4°C to remove cellular debris. Protein concentration was determined using the BCA method.

### Immunoblotting

Sample aliquots containing 10μg of protein were suspended in 1x Laemmli buffer, with or without DTT, boiled at 95°C for 5min and subjected to SDS/PAGE. Proteins were transferred to a nitrocellulose membrane (Bio-Rad, 160112), and incubated with blocking buffer [5% skim milk powder in tris buffered saline with 0.1% tween 20 (TBST)] for 1h at room temperature, followed by overnight incubation at 4°C with primary antibodies in blocking buffer under gentle agitation against p62 (1:2,000, Cell Signaling, 5114s), Parkin (1:2,000, Santa Cruz, sc-32282), LC3I/II (1:2,000, Cell Signaling, 12741s), MFN1/2 (1:2,000, Abcam, ab57602), DRP1 (1:2,000, BD Biosciences 611113), GFER (1:4,000, ProteinTech, 11293-1-AP), COX17 (1:2,000, ProteinTech 11464-1-AP), and Vinculin (1:10,000, Abcam, ab129002). Membranes were washed five times for 5min and probed with an anti-rabbit or anti-mouse IgG conjugated to horseradish peroxidase (HRP) secondary antibody in blocking buffer for 1h at room temperature. Membranes were washed five times for 5min in TBST and protein bands were visualized using the ChemiDoc™ MP Imaging System (Bio-Rad). Densitometry band analysis was performed using Image J software. Abundance of target proteins are presented normalized to Vinculin or Ponceau staining.

### Electron Microscopy

Transmission electron microscopy (TEM) was used to examine the mitochondrial structure in WT and *Grx2^−/−^ tibialis anterior* muscle. In brief, *tibialis anterior* muscle was fixed in 2.5% glutaraldehyde. The tissue was dehydrated in a graded series of ethanol and then embedded in Spurr’s resin. Resin blocks were sectioned using a Leica EM UC6 ultramicrotome (Leica Microsystems). Sections were mounted on copper grids coated with formvar film, stained with 2% alcoholic uranyl acetate and Reynold’s lead citrate and imaged using the JEOL 1230 Transmission Electron Microscope (JEOL Ltd.). Fifty-five micrographs were examined from each genotype at a magnification of 3,000. Quantitative morphometry was then used to determine mitochondrial volume density, as estimated using the point-counting method using Image J software (Zong et al., 2002). Specifically, 10 × 10 grids were overlaid on each of the 55 micrographs per animal, and the intersection point between horizontal and vertical lines were used to determine if intersection point fell with the mitochondria, the cytoplasm, or extracellularly. The percentage of surface area was calculated, and the mean surface area for each animal was used to determine the mean for each group.

### Mitochondrial and Nuclear DNA Quantification

*Tibialis anterior* muscle was homogenized using a Dounce homogenizer with Tris-based lysis buffer [10mM Tris-HCl (pH 8.0), 1mM EDTA, and 0.1% SDS]. Homogenates were incubated with 15mM proteinase K (Invitrogen) at 55°C overnight (Guo et al., 2009). Lysates were vortexed vigorously and the non-soluble fraction was pelleted by centrifugation at 10,000g for 15min at room temperature. DNA was extracted from the supernatant using phenol/chloroform/isoamyl alcohol (25:4:1; PCIAA) as previously described (Guo et al., 2009). qPCR with SYBR Green FastMix (Quanta Biosciences) was run according to the manufacturer’s protocol. The cytochrome c oxidase subunit I (CO1) gene of the mtDNA (F: 5'-TGC TAG CCG CAG GCA TTA C-3' and R: 5'-GGG TGC CCA AAG AAT CAG AAC-3') and the NDUFV1 nDNA gene (F: 5'-CTT CCC CAC TGG CCT CAA G-3' and R: 5'-CCA AAA CCC AGT GAT CCA GC-3') were amplified by qPCR.

### Colocalization Analysis of Mitochondria and Lysosomes

The Mitophagy Detection Kit (Dojindo Molecular Technologies, Rockville, United States) was used according to the manufacturer’s protocol for visualization of mitophagy in primary myoblasts. The kit consists of a Lyso Dye, which stains lysosomes, and a MtPhagy Dye, that accumulates in all mitochondria. Upon acidification due to fusion of the mitochondria to the lysosome, the fluorescent signal of the MtPhagy dye is enhanced to allow for detection of mitophagy (Iwashita et al., 2017). Briefly, 50,000 myoblasts were seeded into four-well Ibidi slides. After 24h, cells were washed with serum-free media, and incubated with the MtPhagy dye for 30min at 37°C. To observe colocalization with lysosomes, cells were washed again with serum-free media and incubated with the Lyso Dye for 30min at 37°C. Following the incubation, cells were washed and imaged with imaging media (phenol red- and serum-free), using a Scientific CMOS Camera using an Olympus 63x/1.4 oil immersion objective with a DeltaVision Elite Microscope (Olympus). Z-stacks were acquired with 1μm spacing and images were deconvolved using the interactive deconvolution in SoftWorx 7.0.

Blinded colocalization analysis between the Lyso dye and MtPhagy dye was performed on images subjected to manual thresholding of the brightest 2% of pixels in each channel images to unbiasedly remove the background (Lorenzen et al., 2010). Images were converted to binary before using the EzColocalization Fiji Plugin (Stauffer et al., 2018). The Mander’s correlation coefficient was calculated to quantify the co-occurrence of signals between the Lyso Dye and MtPhagy Dye, as well as the MtPhagy Dye and Lyso Dye.

### High-Resolution Respirometry in Permeabilized Muscle Fibers

Fibers were prepared from *tibialis anterior* muscle of mice. High-resolution respirometry was conducted using an Oxygraph-2k system (OROBOROS Instruments, Innsbruck, Austria). Dissected *tibialis anterior* samples were placed in ice-cold relaxation BIOPS medium (5.77mM Na_2_ATP, 7.23mM K_2_EGTA, 2.77mMCaK_2_EGTA, 6.56mM MgCl_2_-6H_2_O, 20mM taurine, 60mMK-lactobionate, 15mM phosphocreatine, 20mM imidazole, 0.5mM DTT, and 50mM MES; pH 7.1 at 0°C) immediately after harvesting. Muscle fibers were mechanically separated and permeabilized using saponin (50μg/ml) on ice for 30min. Fibers were then rinsed in mitochondrial respiration medium, MiRO5 (0.5mM EGTA, 3mM MgCl_2_-6H_2_O, 20mM taurine, 10mM K_2_HPO_4_, 20mM HEPES, 110mM sucrose, and 1g/l BSA; pH 7.1 at 37°C), and weighed muscle fibers were placed into the respirometry chambers. Two separate protocols were used and analyses were performed at 37°C. The first protocol included consecutive additions of 2mM malate, 5mM pyruvate, 10mM glutamate, 5mM ADP (complex I-supported respiration), 10mM succinate (complex I- and complex II-supported respiration), 0.25μM titrations of carbonyl cyanide p-trifluoromethoxyphenyl hydrazine (FCCP; maximal respiration) and 2.5μM antimycin A (AMA). The second protocol included successive additions of 2mM malate, 200μM octanoyl carnitine, 5mM ADP (fatty acid-supported respiration), 5mM pyruvate, 10mM glutamate, 10mM succinate, 2.5μM oligomycin (leak respiration). Values are corrected to non-mitochondrial oxygen consumption (i.e., that in the presence of AMA).

### Complex IV Activity

Enzymatic activity of complex IV was determined by quantifying the oxidation of cytochrome C as previously described (Pileggi et al., 2016). In brief, cytochrome C oxidation was quantified at 550nm in 50mM potassium phosphate buffer (pH 7.0), and 50μM of reduced cytochrome C using the BioTek Synergy Mx Microplate Reader (BioTek Instruments, Inc., Winooski, VT, United States). Enzyme activity was calculated using the extinction coefficient *ε* = 18.5, and expressed per μg of protein.

### Identification of Transcript Correlations in the BXD Mouse Tissue Data Sets

Using a BXD mouse genetic reference population (Pirinen et al., 2014), skeletal muscle microarray data (AffymetrixMouseGene1.0 ST) were analyzed for transcript expression correlations between *Grx2* and genes associated with the disulfide relay system using the GeneNetwork program.^1^

### Statistical Analysis

Statistical analyses were performed using Prism software (GraphPad). Statistical significance between WT and *Grx2^−/−^* mice was determined using a two-tailed *t*-test. For experiments involving colchicine treatment, statistical significance was determined using two-way ANOVA with genotype and colchicine as factors. Pairwise comparisons were determined using the Holm-Sidak *post hoc* method. Data are shown as means ± SEM. Statistical significance was accepted at *p* < 0.05. Prism software (GraphPad) was used to generate graphs.

## Results

### Grx2 Deficiency Results in Decreased Lean Mass and Skeletal Muscle Cell Cross-Sectional Area

**Figure 1:**
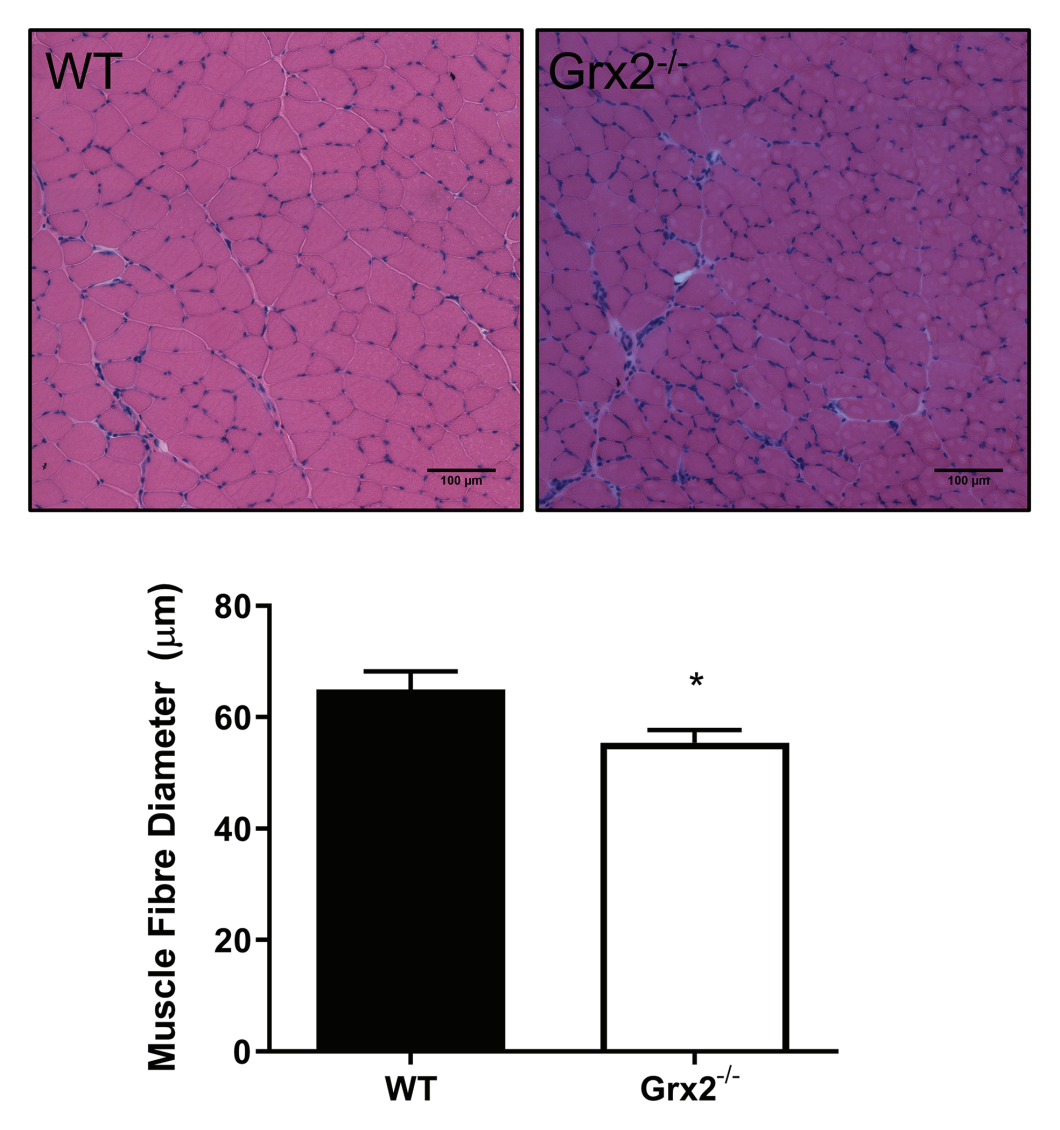
Skeletal muscle fiber diameter is decreased in tibialis anterior muscle in *Grx2^−/−^* mice. Muscle fiber diameter was determined in cross-sections of frozen optimal cutting temperature (OCT)-embedded *tibialis anterior* muscle. *Grx2^−/−^* mice displayed decreased muscle fiber diameter compared to wild-type (WT) mice. Representative images are shown above the graph. Data are expressed as mean ± SEM. *n* = 5, *p < 0.05.

Previously, we demonstrated that *Grx2^−/−^* mice aged between 9 and 12 weeks exhibit normal linear growth with no differences in liver, skeletal muscle, and brown adipose tissue weights (Mailloux et al., 2014). We documented hypertension even in young male *Grx2^−/−^* mice, despite no evidence of a cardiac phenotype. In the current study, we did not assess blood pressure, but it is possible that hypertension may be contributing overall to our current findings. Therefore, we aimed to further investigate body composition and metabolic phenotyping, and to focus on young male *Grx2^−/−^* mice (4–6weeks) when there is no evidence of heart pathology (e.g., hypertrophy), which develops at ~10weeks of age. We first measured body weights and body composition (Echo-MRI) and found that total body weight and lean mass were slightly decreased in the *Grx2^−/−^* compared to WT mice (*p* < 0.01 for body weight and lean mass; Table 1). In contrast, there were no differences in total body fat mass between the two genotypes (Table 1). *Ad libitum* food intake was measured in the 12 chamber, fully automated CLAMS system and was found not to differ between genotypes (Table 1). VO_2_ consumption rates normalized to lean body mass were similar between WT and *Grx2^−/−^* mice (Table 1). Similarly, the RER (VCO_2_/VO_2_), which indicates the relative proportion of fuels being oxidized at the whole-body level (i.e., carbohydrates vs. fats) did not differ between genotypes (Table 1). We next examined the cross-sectional area of H&E stained skeletal muscle sections to determine if differences in skeletal muscle size were contributing to the decrease in lean body mass in *Grx2^−/−^* mice. We conducted these and subsequent analyses in *tibialis anterior* muscles, which are known to be composed of predominantly glycolytic fibers, with a mix of oxidative and intermediate fiber types (Augusto et al., 2004). *Tibialis anterior* sections from *Grx2^−/−^* mice exhibited a smaller myofiber diameter than WT mice (*p* < 0.05, Figure 1).

**Table 1:** *Grx2*^−/−^ mice weigh less and have decreased lean muscle mass despite equal activity and food intake.

	WT	*Grx2^−/−^*	*p*-value
Body weight (g)	21.12 ± 0.32	19.66 ± 0.22^*^	*p* < 0.01
Tibialis anterior mass (mg)	38.46 ± 0.78	37.18 ± 1.03	*p* = 0.35
Gastrocnemius mass (mg)	252.68 ± 9.81	209.45 ± 14.53^*^	*p* = 0.03
Fat mass (g)	1.26 ± 0.09	1.28 ± 0.11	*p* = 0.88
Fat free mass (g)	18.34 ± 0.38	16.53 ± 0.44^*^	*p* < 0.01
Food intake	7.68 ± 1.42	9.59 ± 2.58	*p* = 0.53
Activity-light cycle (wheel count)	17,727.21 ± 6362.12	9,429.98 ± 3,215.67	*p* = 0.76
Activity-dark cycle (wheel count)	22,687.83 ± 8,612.37	12,131.72 ± 3,911.19	*p* = 0.59
VO_2_/lean mass-light [(ml/kg/h)/kg]	3.88 ± 0.19	4.02 ± 0.11	*p* = 0.94
VO_2_/lean mass-dark [(ml/kg/h)/kg]	5.05 ± 0.26	5.00 ± 0.73	*p* = 0.99
RER light	0.91 ± 0.01	0.88 ± 0.01	*p* = 0.38
RER dark	0.95 ± 0.01	0.94 ± 0.01	*p* = 0.93

* *p* < 0.05 WT vs. *Grx2*^−/−^, *n* = 11 per genotype.

Data are expressed as means ± *SEM*.

### Absence of Grx2 Perturbs Muscle Glutathione Redox

**Figure 2:**
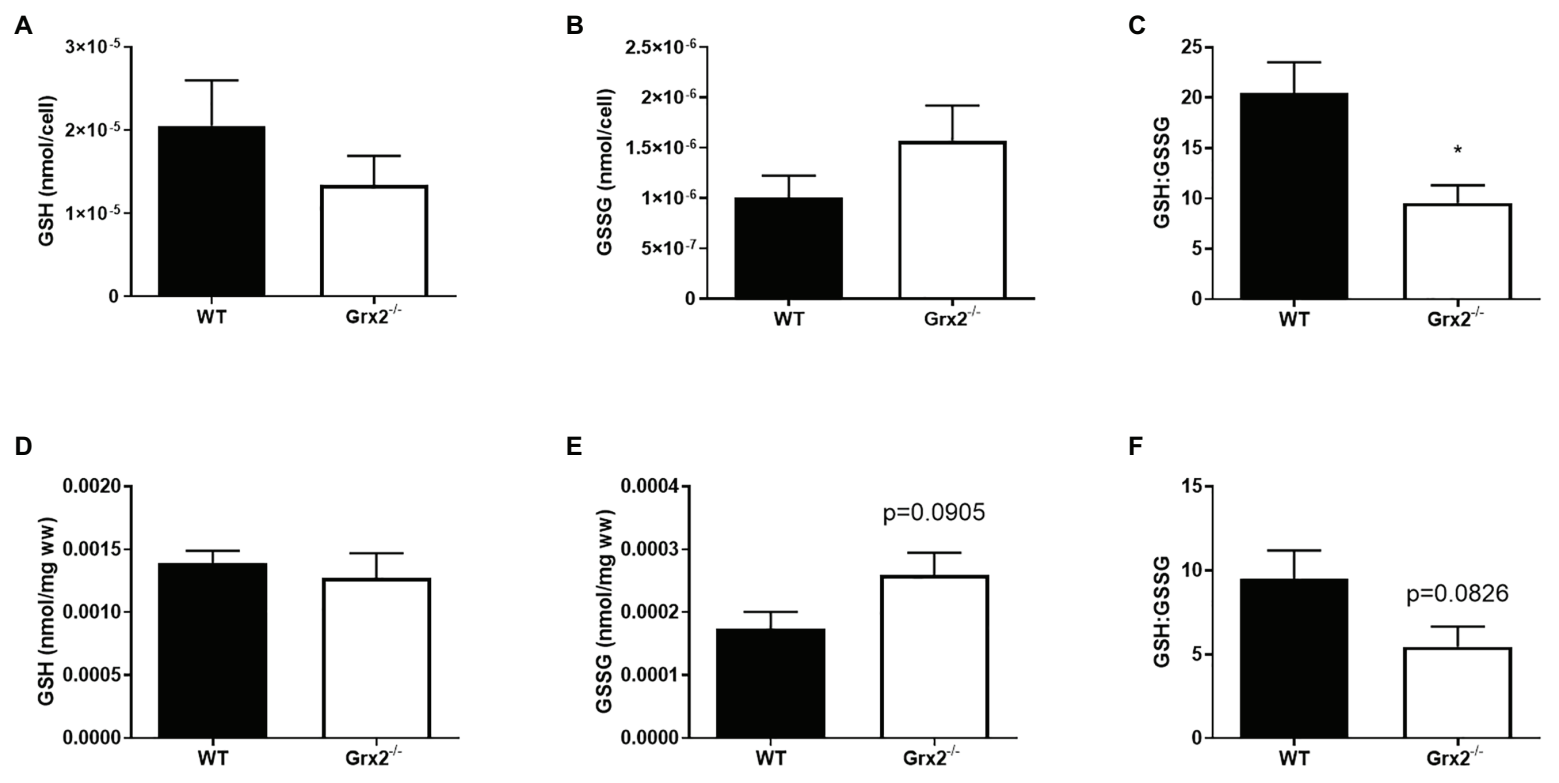
GSH:GSSG is decreased in *Grx2^−/−^* myoblasts. High-performance liquid chromatography (HPLC) analyses were used to determine the concentrations of reduced glutathione (GSH), and oxidized glutathione (GSSG) in primary myoblasts and *tibialis anterior* muscle. **(A)** GSH and **(B)** GSSG did not differ between WT and *Grx2^−/−^* primary myoblasts. **(C)** GSH:GSSG is decreased in *Grx2^−/−^* myoblasts compared to their WT counterparts. **(D)** Absolute concentrations of GSH did not differ between genotypes in *tibialis anterior* muscle. **(E)** There was a trend for increased GSSG in *Grx2^−/−^* muscle. **(F)** GSH:GSSG tended to decrease in *Grx2^−/−^* muscle. Data are mean ± SEM. *n* = 6–7 for determinations in primary myoblasts **(A-C)**; *n* = 5 for determinations in in *tibialis anterior* muscle **(D-F)**; *p < 0.05.

*Grx2* plays an important role in regulating protein (de)glutathionylation reactions of mitochondrial target proteins during oxidative stress. Absence of *Grx2* results in a decreased GSH:GSSG ratio in isolated mitochondria from skeletal muscle (Mailloux et al., 2013) and the accumulation of GSSG has been linked to molecular adaptions that favor mitochondrial fusion during cellular stress (Shutt et al., 2012). Therefore, we measured GSH in primary myoblasts from within *Grx2^−/−^* mice to potentially confirm the finding of GSSG, and to investigate the effects of altered GSH redox on mitochondrial fusion. Our findings revealed that absolute amounts of GSH and GSSG per cell did not differ between genotypes (Figures 2A,B) in primary myoblasts. However, *Grx2^−/−^* myoblasts had a significantly decreased glutathione redox ratio when compared to their WT counterparts (*p* < 0.01, Figure 2C). Next, we aimed to confirm our findings in Grx2^−/−^ *tibialis anterior* tissue. Our results demonstrate that Grx2^−/−^ muscle tended to have increased concentrations of GSSG (*p* = 0.0905, Figure 2E), whereas the concentration of GSH did not differ between genotypes (Figure 2D). The increase in GSSG in Grx2^−/−^ muscle was reflected by a trend for decreased glutathione redox ratio in the *tibialis anterior* from Grx2^−/−^ mice (*p* = 0.0826, Figure 2F).

### Deletion of Grx2 Promotes Mitochondrial Fusion

**Figure 3:**
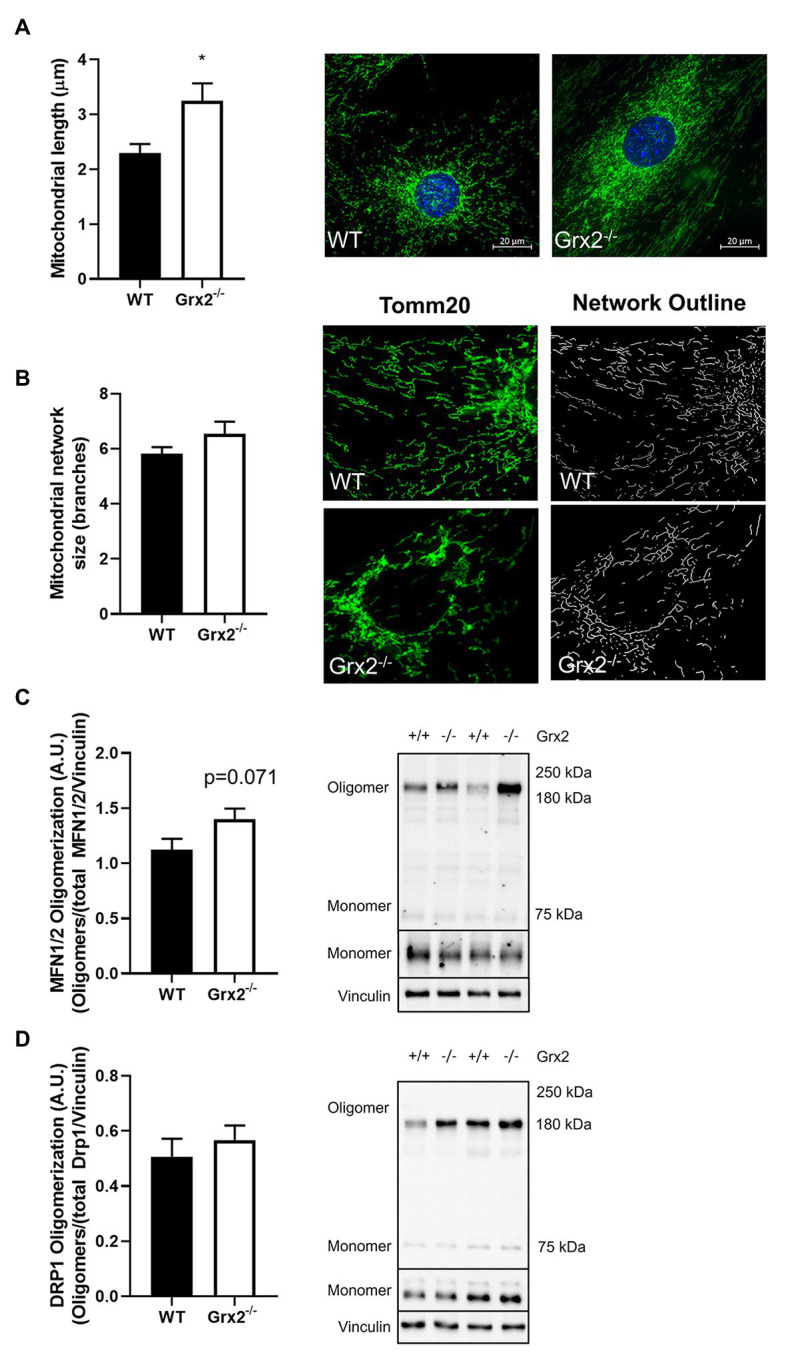
Mitochondrial length is increased in *Grx2^−/−^* myoblasts. **(A)** Mitochondrial length is increased by ~30% in *Grx2^−/−^* myoblasts. Representative images of WT and *Grx2^−/−^* myoblasts are shown beside the graph; scale bars, 20μm. Quantification of 50 mitochondrial lengths from five independent experiments. **(B)** Mitochondrial branching does not differ between WT and *Grx2^−/−^* myoblasts. Representative images of WT and *Grx2^−/−^* myoblasts are shown **(C)** Oligomerization of MFN1/2 tends to be increased in *Grx2^−/−^* skeletal muscle (*p* = 0.071). **(D)** Oligomerization of DRP1 in skeletal muscle does not differ between WT and *Grx2^−/−^.* Data are expressed as mean ± SEM. *n* = 6–7 for mitochondrial length determinations in primary myoblasts **(A)**; *n* = 5 for for mitochondrial branching analysis **(B)**; *n* = 6 for quantification of MFN1/2 and DRP1 oligomerization in *tibialis anterior* muscle **(C,D)**; *p < 0.05.

Following the confirmation of a decreased GSH:GSSG in *Grx2^−/−^* primary myoblasts, we hypothesized that mitochondria in primary muscle cells of *Grx2^−/−^* mice would show increased fusion compared to muscle cells of WT mice. Quantitative morphometric analyses of TOM20-stained primary myoblasts from both genotypes showed that mitochondria were indeed more fused, as evidenced by an increased mitochondrial length in *Grx2^−/−^* vs. WT myoblasts (*p* < 0.05, Figure 3A). When assessing mitochondrial network complexity, we did not observe an increase in mitochondrial branching in *Grx2^−/−^* primary myoblasts (*p* = 0.18, Figure 3B). Next, to examine whether the increase in mitochondria length was attributable to redox control of the formation of MFN oligomer complexes, we immunoblotted for MFN1/2 and DRP1 under non-reducing conditions to preserve disulfide bonds. There was a trend for increased content of higher order MFN1/2 oligomers between 160 and 250kDa (*p* = 0.071, Figure 3C). In contrast, there was no difference in the formation of DRP1 oligomers (Figure 3D). Given that we observed MFN1/2 and DRP1 high molecular weight (MW) oligomers by western blot only under non-reducing conditions, we conclude that these high MW oligomers are similarly disulfide-mediated and would reduce in response to redox potential.

### Mitochondrial Ultrastructure and Autophagy Are Altered in Grx2 Deficient Muscle

**Figure 4:**
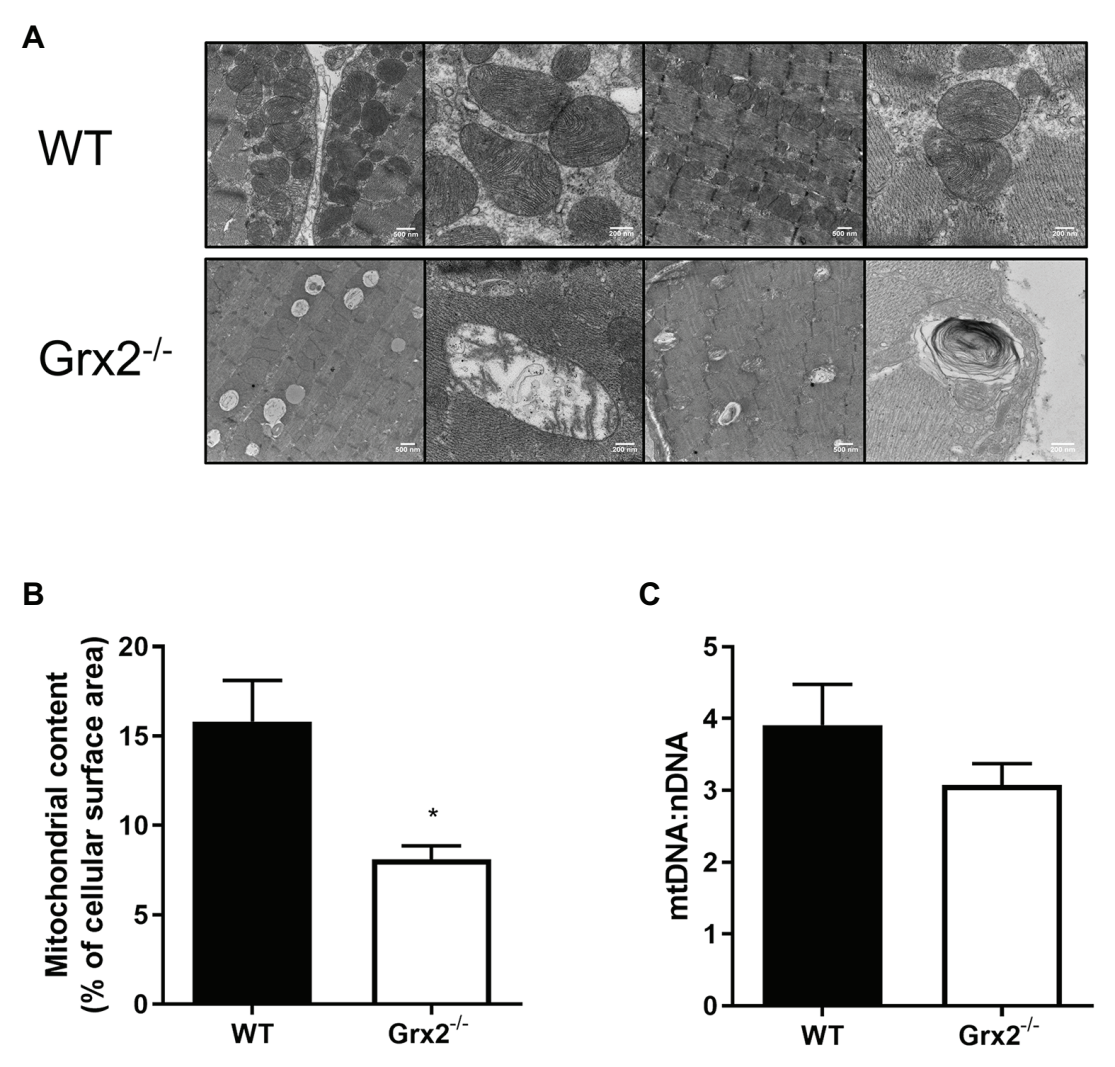
Electron micrographs show disordered mitochondrial morphology and ultrastructure, and a decrease in mitochondrial volume in *Grx2^−/−^* skeletal muscle. **(A)** Representative micrographs of WT and *Grx2^−/−^ tibialis anterior* muscle. WT mitochondria have well-defined cristae structures with normal mitochondrial shape. In contrast, in *Grx2^−/−^ tibialis anterior* there are vacuole-like structures with double membranes, and abnormal mitochondrial cristae. **(B)** Quantitative analyses of images revealed decreased mitochondrial surface area in *Grx2^−/−^*. **(C)** No differences were found in the ratio of mtDNA to nDNA. Data are expressed as mean ± SEM. *n* = 3 for TEM analysis **(A,B)**; *n* = 6 for quantification of mtDNA:nDNA **(C)**; *p < 0.05.

Following our findings of elevated mitochondrial fusion in primary *Grx2^−/−^* myoblasts, we then examined mitochondrial ultrastructure in skeletal muscle tissue using TEM to observe the effects of *Grx2* deficiency *in vivo*. Electron micrographs showed that mitochondrial morphology is markedly abnormal in the *Grx2^−/−^* muscle (Figure 4A). The WT mitochondria cristae were found to be well-defined, and tubular. However, micrographs showed that *Grx2^−/−^* mitochondria frequently lacked ordered cristae (Figure 4A) and had unusual vacuole-like structures with double membranes that resemble mitochondria engulfed in autophagosomes, consistent with the possibility of impaired mitophagy (Ding and Yin, 2012; Menges et al., 2017; Chakraborty et al., 2020). Furthermore, quantitative morphometric analyses revealed that mitochondrial content in the *Grx2^−/−^* muscle was less than half that in the WT skeletal muscle (Figure 4B). We then sought to confirm this by using a common proxy method to measure mitochondrial content, the ratio of mitochondrial DNA to nuclear DNA (mtDNA:nDNA); however, no differences were observed between the WT and *Grx2^−/−^* mtDNA:nDNA (Figure 4C).

**Figure 5:**
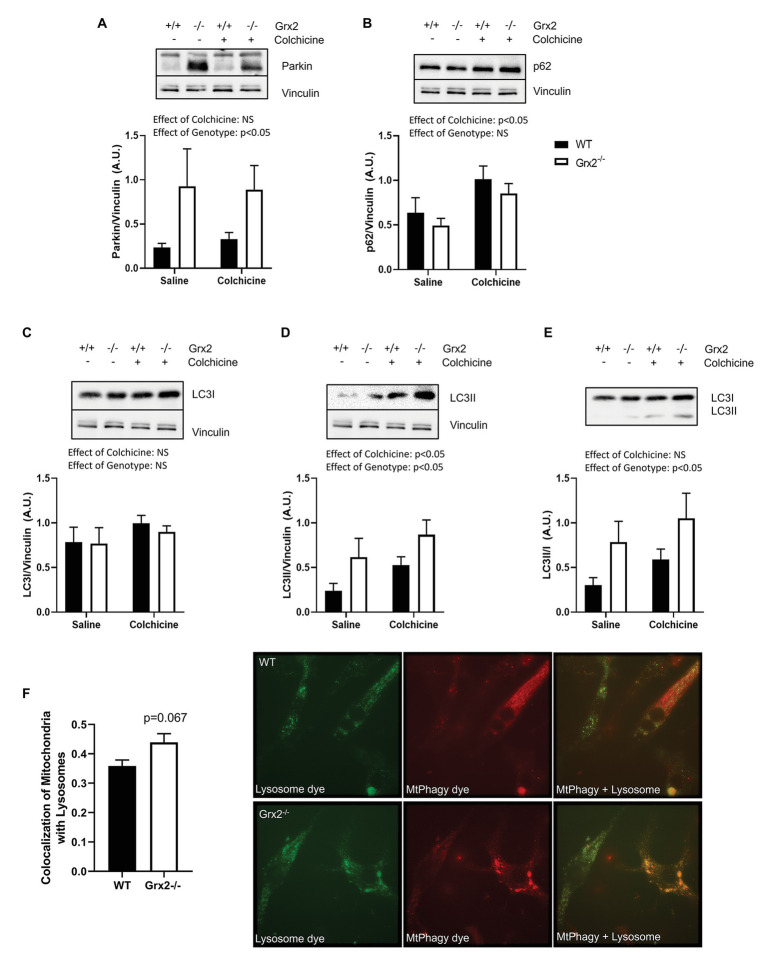
*Grx2^−/−^* skeletal muscle has elevated autophagic flux. Immunoblot analyses was performed on *tibialis anterior* muscle; protein was extracted from colchicine injected mice. **(A)** Protein expression of parkin is elevated in *Grx2^−/−^* muscle. **(B)** Expression of p62 does not change in the absence of *Grx2*, but increases with colchicine treatment. **(C)** LC3I expression does not differ between genotypes or with colchicine treatment. **(D)** LC3II protein expression is increased with colchicine treatment and in *Grx2^−/−^* muscle. **(E)** Autophagic flux is elevated in *Grx2^−/−^* muscle as determined by LC3II/I. Equal protein loading was determined by immunoblotting for vinculin. Data are expressed as mean ± SEM, *n* = 6–7. Representative blots are shown above the graphs. **(F)** *Grx2^−/−^* primary myoblasts exhibit increased colocalization of mitochondria and lysosomes. Colocalization of mitochondria and lysosomes in primary myoblasts using Mander’s correlation coefficient (0 = no colocalization, 1 = full colocalization). Representative images of WT and *Grx2^−/−^* myoblasts. Quantification of 6–10 blinded images per animal. Data expressed as mean ± SEM, *n* = 4.

We then hypothesized that the irregular ultrastructure of the mitochondria and the multi-lamellar structures in the *Grx2^−/−^* skeletal muscle may be indicative of increased autophagy of mitochondria, and that the mtDNA may be retained within autophagosomes. Therefore, we immunoblotted for key proteins involved in autophagy and mitochondrial clearance in skeletal muscle homogenates from mice that were injected intraperitoneally with colchicine or an equal volume of saline for 2days prior to sacrifice (Vainshtein et al., 2015). Autophagic flux is determined by quantifying protein levels in the presence and absence of chemical agents such as colchicine, that destabilize microtubules and block lysosomal degradation (Ju et al., 2010). Immunoblotting for key proteins involved in the priming of autophagic and mitophagic pathways revealed that protein expression of parkin was elevated in *Grx2^−/−^* muscle (main effect of genotype *p* < 0.05; Figure 5A). In contrast, while colchicine treatment promoted increased expression of p62, the expression of p62 did not differ between genotypes (main effect of colchicine *p* < 0.05; Figure 5B). Expression of the downstream autophagic precursor, LC3I, did not differ between genotypes or with colchicine treatment (Figure 5C), whereas there was a main effect for increased LC3II expression for both colchicine treatment (main effect of colchicine *p* < 0.05) and absence of *Grx2* (main effect of genotype *p* < 0.05; Figure 5D). The ratio of LC3II/I is used as a quantitative indicator of autophagic flux (Kadowaki and Karim, 2009). LC3II/I was elevated in the *tibialis anterior* of *Grx2^−/−^* mice (main effect of genotype *p* < 0.05; Figure 5E). Next, to confirm our findings, we used a commercially available mitophagy kit to quantify the colocalization of mitochondria and lysosomes in primary myoblasts. Blinded analysis of acquired images revealed a trend for increased colocalization between mitochondria and lysosomes in *Grx2^−/−^* myoblasts compared to WT (*p* = 0.067, Figure 5F).

### Complex I Mitochondrial Respiration Is Impaired in Grx2 Deficient Skeletal Muscle

**Figure 6:**
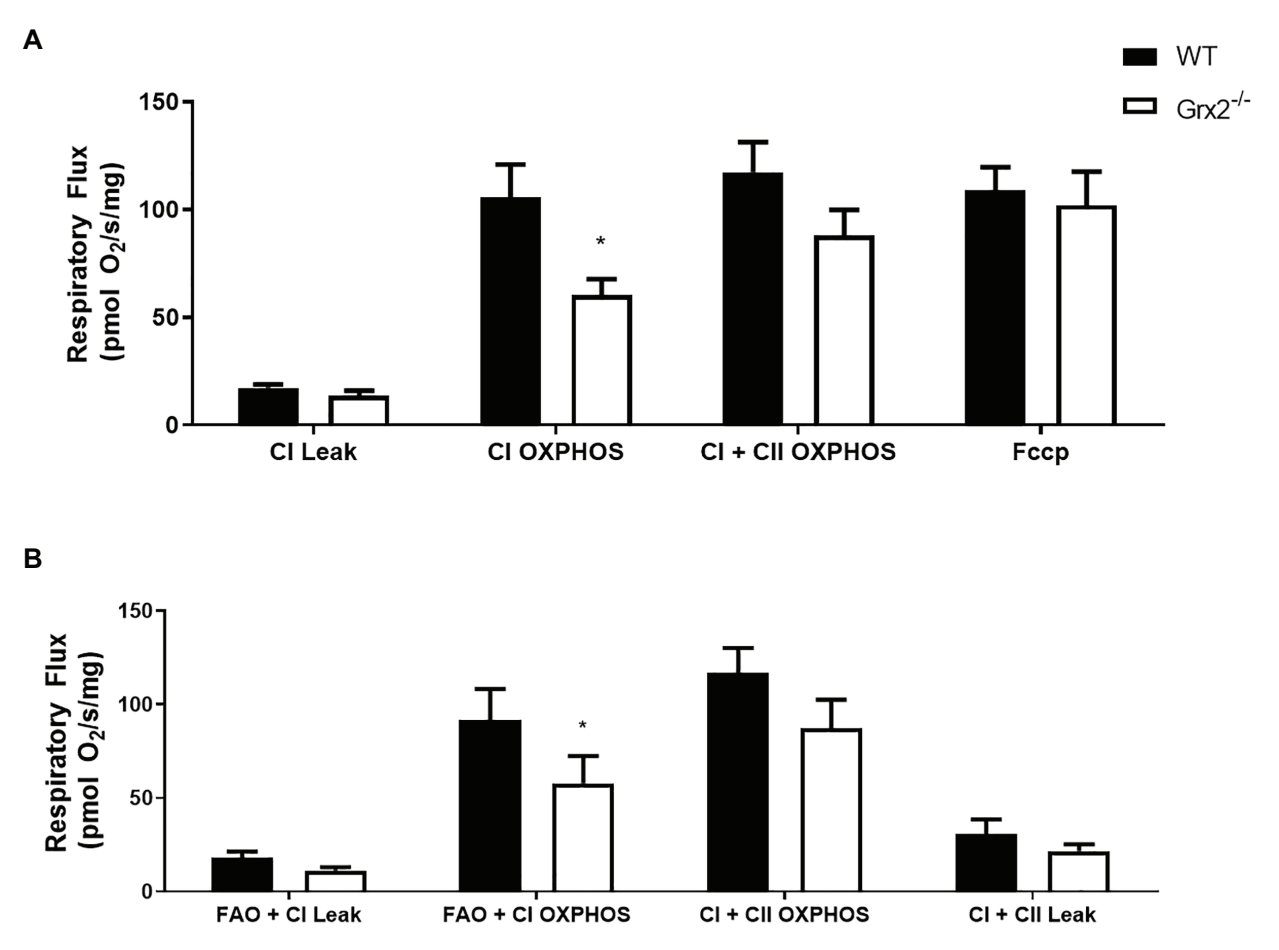
Mitochondrial complex I (CI) oxidative phosphorylation (OXPHOS) is impaired in permeabilized *Grx2^−/−^* mouse muscle. **(A)** Respiratory flux per milligram of saponin-permeabilized *tibialis anterior* muscle demonstrates that CI OXPHOS is impaired in *Grx2^−/−^* mice using the substrates pyruvate, malate, and glutamate. **(B)** Complex I respiration using octanoylcarnitine, in combination with malate, pyruvate, and glutamate is also decreased in *Grx2^−/−^* muscle. Data are expressed as mean ± SEM. *n* = 6; *p < 0.05.

Subsequent to determining that mitochondrial structure was disordered in skeletal muscle, we sought to examine the functional effects of *Grx2* deficiency on skeletal muscle mitochondrial bioenergetics in permeabilized muscle fibers. Previously, our investigations into the effects of *Grx2* deficiency in skeletal muscle were conducted on mitochondrial isolations from homogenized muscle (Mailloux et al., 2014), in which the isolation process may exclude damaged mitochondria resulting in the preferential selection of healthy mitochondria subpopulations (Piper et al., 1985). Thus, we used published protocols to permeabilize the sarcolemma and examine mitochondrial oxidative phosphorylation (OXPHOS) *in situ* using high-resolution respirometry. Our results demonstrate impaired complex I (CI) OXPHOS in *Grx2^−/−^* permeabilized myofibers (Figure 6A; *p* < 0.05). Furthermore, maximal respiration, measured by the addition of the uncoupler FCCP (F), was not different between genotypes. Notably, FCCP titrations increased respiration in *Grx2^−/−^* muscle, but not WT muscle. Occasionally FCCP titrations do not result in an additional increase in respiratory flux (Christiansen et al., 2015), often due to saturating concentrations of substrates, inhibition of oxygen consumption associated with the use of high FCCP concentrations, or from the use of sub-optimal FCCP concentrations resulting in non-maximal stimulation (Steinlechner-Maran et al., 1996; Pesta and Gnaiger, 2012). To determine if there were OXPHOS defects when respiration was fueled by fatty acid oxidation (FAO), we used a separate protocol using octanoyl carnitine in additional samples of myofibers. FAO+CI OXPHOS was again found to be decreased (Figure 6B).

### Grx2 Deletion Is Associated With Dysregulation of the Disulfide Relay System

**Figure 7:**
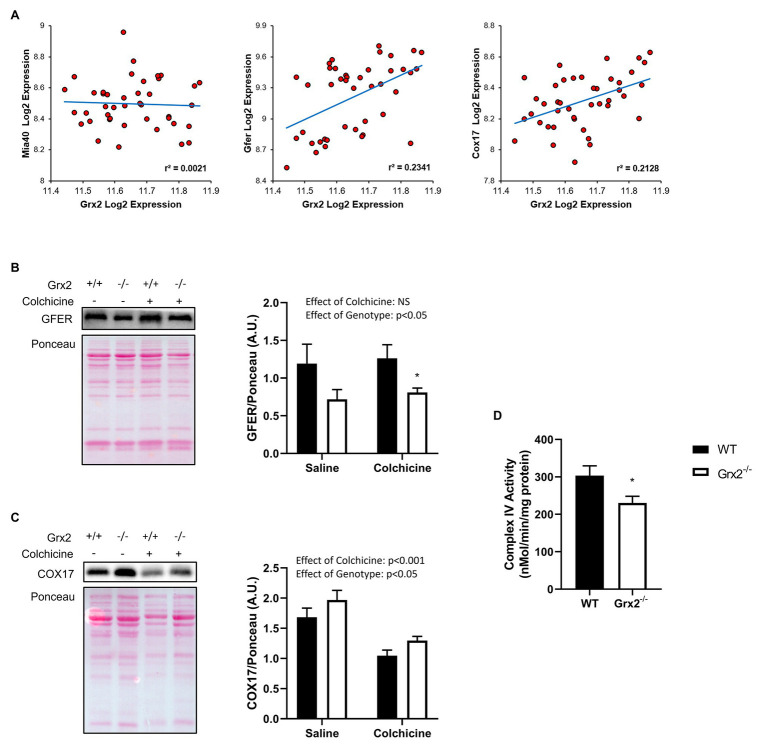
Grx2 deficiency is associated with dysregulation of the disulfide relay system. **(A)** Genes involved in the disulfide relay system were correlated to *Grx2* using 42 BXD mouse strains fed a chow diet. *Grx2* is positively correlated with *Gfer* and *Cox17* in skeletal muscle. **(B)** *Tibialis anterior* muscle protein was extracted from saline and colchicine injected mice, and protein expression level of GFER was lower in *Grx2^−/−^* skeletal muscle, as determined by western blot. **(C)** Protein expression of COX17 was increased in *Grx2^−/−^*, and decreased in skeletal muscle from mice treated with colchicine. Equal protein loading was determined with Ponceau stain. **(D)** Complex IV activity is decreased in skeletal muscle from *Grx2^−/−^* mice. Data are expressed as mean ± SEM. *n* = 6–7 for GFER and COX17 quantification; *n* = 6 for complex IV activity. *p < 0.05.

Lastly, we sought to determine the effects of *Grx2* deletion on components of the disulfide relay system. Most nuclear-encoded mitochondrial proteins are synthesized as precursors on cytosolic ribosomes, and subsequently imported *via* translocases on the mitochondrial outer membrane to the IMS to be directed towards protein folding pathways (Neupert and Herrmann, 2007). A subset of cysteine-rich proteins rely on the disulfide relay system for their retention in the IMS, in which Mia40 (IMS Import and Assembly) binds to cysteine residues on precursor proteins to facilitate the formation of a mixed disulfide bond (Mesecke et al., 2005). The disulfide complex is resolved with the oxidation of the substrate, resulting in the reduction of Mia40. Mia40 is subsequently recycled through oxidation by the growth factor, augmenter of liver regeneration (GFER, ERV1 homolog in Yeast; Mesecke et al., 2005; Hell, 2008; Lionaki et al., 2010; Kojer et al., 2012). GFER is subsequently re-oxidized through electron transfer to cytochrome c, which is recycled through the activity of cytochrome c oxidase (Allen et al., 2005). *Grx2* is thought to play a key role in maintaining the redox state of the IMS, as *Grx2* is dually localized in the mitochondrial IMS and matrix (Kojer et al., 2015). Moreover, overexpression of *Grx2* delays protein folding by maintaining Mia40 in a reduced state (Kojer et al., 2015). As complex IV function is essential to the function of the disulfide relay system, and complex IV assembly proteins COX17 and COX19 are known substrates of this pathway (Mesecke et al., 2005; Gabriel et al., 2007; Hell, 2008), we hypothesized that the deletion of *Grx2* would alter complex IV activity. Therefore, we examined the correlation between *Grx2* gene expression and key genes involved in the disulfide relay system using BXD mice. Here, we show that *Grx2* is positively correlated with *Gfer* (Figure 7A, Pearson correlation coefficient = 0.484, *p* < 0.001) and *Cox17* gene expression (Figure 7A, Pearson correlation coefficient = 0.461, *p* < 0.001). Next, we quantified the protein expression of GFER and Cox17 in *Grx2^−/−^* skeletal muscle, and determined complex IV activity. Consistent with our hypothesis, GFER expression was decreased in *Grx2^−/−^* skeletal muscle (Figure 7B; Main effect of genotype *p* < 0.05), independent of colchicine treatment. In contrast, COX17 protein expression was increased in *Grx2^−/−^* skeletal muscle (Figure 7C; Main effect of genotype *p* < 0.05), and decreased with colchicine treatment (Figure 7C; Main effect of colchicine *p* < 0.001). Complex IV activity was decreased in *Grx2^−/−^* skeletal muscle (Figure 7D, *p* < 0.05).

## Discussion

**Figure 8:**
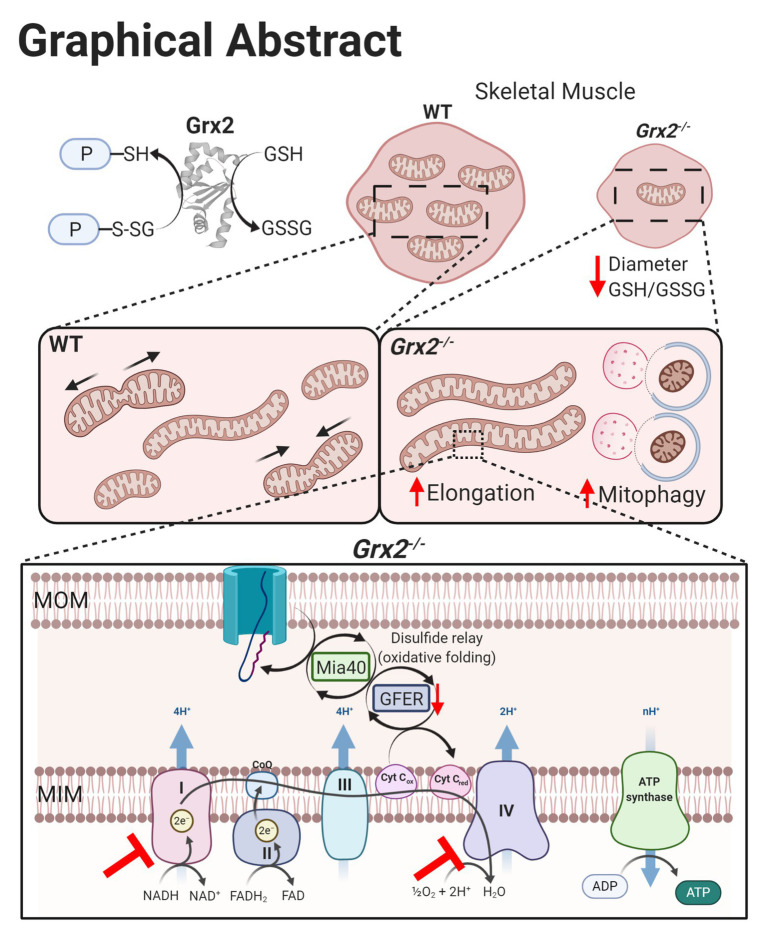
Graphical summary of effects on skeletal muscle following *Grx2* deletion. Glutaredoxin-2 (Grx2) is a glutathione-dependent oxidoreductase that reversibly (de)glutathionylates sulfhydryl residues on mitochondrial proteins, thereby directly maintaining the pool of oxidized and GSH and redox homeostasis. Deletion of *Grx2* alters skeletal muscle morphology, mitochondrial ultrastructure, and activity. Myofiber diameter of *tibialis anterior* muscle is smaller following *Grx2* deletion, and the ratio of reduced to oxidized glutathione is decreased. The mitochondrial network is elongated in *Grx2^−/−^* primary myoblasts and TEM images of *Grx2^−/−^* myofibers reveal mitochondria encapsulated in vacuole-like structures resembling autophagosomes. Lastly, the oxidative folding pathway and the electron transfer system are dysregulated as a consequence of *Grx2* deletion, consistent with the overall importance of Grx2 in maintaining mitochondrial protein structure and function. Graphical abstract was created with BioRender.com.

The present study examined the impact of *Grx2* deficiency on skeletal muscle mitochondrial ultrastructure and dynamics, as well as on bioenergetics in *ex vivo* muscle fibers. Our results demonstrate that *Grx2* is involved in maintaining mitochondrial ultrastructure by regulating fusion in primary myoblasts. We provide evidence that deletion of *Grx2* perturbs muscle glutathione redox status, resulting in increased autophagic flux and the formation of abnormal autophagic vacuoles. These structural abnormalities were accompanied by decreased complex I –supported OXPHOS activity in muscle fibers. Taken together, findings indicate that *Grx2* controls skeletal muscle mitochondrial fusion, ultrastructure, and complex I-driven respiration (Figure 8).

Mitochondrial morphology is thought to alter energy transduction, as enhanced fusion is often associated with increased mitochondrial function (Liu et al., 2009; Zick et al., 2009). Mitochondria can also elongate as an acute stress-response to remain protected from autophagic degradation and activation of apoptosis (Tondera et al., 2009; Gomes et al., 2011). Mitochondrial remodeling can occur in response to changes in cellular redox, and the accumulation of GSSG, in particular, has been linked to mitochondrial elongation (Shutt et al., 2012). Specifically, Shutt et al. (2012) demonstrated that a decrease in the GSH:GSSG ratio induces mitochondrial elongation *via* priming of fusion machinery and generation of MFN disulfide-mediated oligomers. In the context of the current study, our findings of perturbed glutathione redox in *Grx2* deficient primary myoblasts, as indicated by a decrease in the ratio of GSH:GSSG, may contribute to the observed mitochondrial elongation in *Grx2^−/−^* myoblasts. Furthermore, the trend for increased oligomerization of MFN1/2 in *Grx2^−/−^* muscle supports the idea that the coil-coil heptad repeat domains (HR2) domains of MFN1/2 contain cysteine residues located in the intermembrane space that are partially regulated by redox signaling (Mattie et al., 2018). Mitochondrial hyperfusion is generally thought to be a nullifying response to cellular stress; however, prolonged hyperfusion may lead to the accumulation of damaged mitochondrial proteins and activation of autophagy (El Fissi et al., 2018).

Alterations of mitochondrial topology are commonly observed in diseases associated with impaired mitochondrial function (Chan, 2020). Here we show profoundly abnormal mitochondrial ultrastructural characteristics, including decreased mitochondrial surface area and the appearance of vacuole-like double membranous structures. The abnormal mitochondrial ultrastructure observed in the Grx2^−/−^ skeletal muscle is consistent with results from livers dissected from mice treated with ethanol or acetaminophen (Ding and Yin, 2012), and mouse embryonic fibroblasts (MEFS) and SH-SY5Y neuroblasts treated with CCCP (Miyazono et al., 2018; Chakraborty et al., 2020). The decrease in mitochondrial surface area is suggestive of a decrease in mitochondrial density, however analysis of mtDNA:nDNA copy number did not reveal differences between genotypes. Abnormal onion-like structures, similar to the membranous structures detected in the *Grx2^−/−^* muscle, have been observed in OPA1 and mitofilin-depleted cells (Olichon et al., 2003; John et al., 2005). The multi-lamellar membranous structures are thought to be the result of lysosomal contact with mitochondria (Wong et al., 2018). Interestingly, similar membranous structures were observed in neurons from *Drosophila* with a Charcot-Marie-Tooth disease type 2A (CMT2A) MFN2 mutation (R364W^like^) that results in mitochondrial hyperfusion (El Fissi et al., 2018). Similar to our findings in *Grx2^−/−^* muscle, of mtDNA copy number did not differ in R364W^like MFN2^ mutant brains, but rather the mtDNA mutation load was elevated (El Fissi et al., 2018). Therefore, it is possible that mtDNA is retained in the membranous onion-like structures, and subjected to damage. Within the context of the current study, the decreased mitochondrial surface area, combined with the presence of abnormal double membranous structures, are consistent with increased autophagy in *Grx2^−/−^* muscle.

Autophagy is a highly conserved process that involves the lysosomal degradation of cellular components sequestered in autophagosomes. ROS can induce autophagy as a mechanism to combat oxidative stress and damage, and the activity of autophagy-related protein (ATG)-4 is regulated by the reversible oxidation of Cys81 (Scherz-Shouval et al., 2007; Scherz-Shouval and Elazar, 2011). More recently, glutathione redox has been implicated in the activation of autophagy, with initial evidence demonstrating that the depletion of GSH activates mitophagy in yeast (Deffieu et al., 2009). In mammalian systems, glutathione depletion has been shown to activate autophagy in carcinoma cells (Desideri et al., 2012) and retinal pigment cells (Sun et al., 2018). Thus, our finding of increased autophagic flux in muscle of *Grx2^−/−^* mice, indicated by elevated LC3II/I, supports a role for glutathione redox regulation of autophagy. Moreover, the trend for increased colocalization of mitochondria and lysosomes in *Grx2^−/−^* primary myoblasts is consistent with increased mitophagy. The removal of mitochondria by mitophagy appears to be occurring through the traditional PINK1/parkin pathway, which relies on parkin to polyubiquitinate outer membrane target proteins to encapsulate mitochondria in the autophagosome, as parkin expression was elevated in *Grx2^−/−^* skeletal muscle from mice injected with saline and colchicine. Other proteins in the Bcl-2 family can also interact directly with LC3-II to encapsulate mitochondria independent of parkin-mediated ubiquitination, and may contribute to the induction of mitophagy in *Grx2^−/−^* skeletal muscle. Altogether, these findings of increased autophagic flux and appearance of multi-lamellar membranous structures in *Grx2^−/−^* skeletal muscle support a role for the redox control of autophagy.

Reversible glutathionylation reactions play important roles in controlling mitochondrial redox, as mitochondria are a major source of superoxide (Turrens, 1997). Protein thiols on specific subunits of complex I (Ndusf1, Ndufv1, and ND3; Beer et al., 2004), are known to be particularly susceptible to regulation by glutathionylation (Cadenas and Davies, 2000). Here, we demonstrate that complex I-supported respiration was decreased in *Grx2^−/−^* permeabilized skeletal muscle fibers. In line with these findings, we have previously shown that glutathionylation of complex I subunits, is regulated by *Grx2* in cardiac tissue (Mailloux et al., 2014). Modifications to complex I thiols decrease complex I activity in isolated mitochondria from the brain (Balijepalli et al., 1999) and liver (Mailloux et al., 2013). Moreover, deletion of *Grx2* in human lens epithelial cells and in primary neonatal cardiomyocytes results in decreased complex I activity and basal mitochondrial function, respectively (Wu et al., 2010; Kanaan et al., 2018). Thus, our current findings, support a role for the redox control of complex I by glutathionylation actions of *Grx2*.

The mitochondrial disulfide relay system is responsible for the retention and oxidative folding of cysteine-rich proteins in the IMS (Mesecke et al., 2005). Moreover, the disulfide relay system is intricately linked with the mitochondrial electron transport system, as reduced ERV1 (GFER homolog in yeast) can transfer electrons cytochrome C and subsequently cytochrome c oxidase (Complex IV) to induce ERV1 oxidization (Dabir et al., 2007). *Grx2* is dually localized in the IMS and matrix of the mitochondria, and is thought to play a regulatory role in the recycling of ERV1/GFER by regulating glutathione redox in the IMS (Kojer et al., 2012, 2015). In yeast, overexpression of *Grx2* delays protein folding by disrupting the oxidation of Mia40 by ERV1 (Kojer et al., 2015). The absence of *Grx2* also impairs oxidative protein folding in yeast, a consequence attributed to the proofreading actions of glutathione in oxidized protein folding, in which GSH prevents the formation of partially oxidized intermediates to ensure complete and efficient substrate oxidation (Bien et al., 2010). Our findings support the notion that *Grx2* may play a proofreading role in the disulfide relay system (Kojer et al., 2012, 2015). Our correlation analyses revealed that *Grx2* is positively associated with Gfer and Cox17 and we found that deletion of *Grx2* in skeletal muscle results in decreased GFER protein expression, lower complex IV activity, and increased expression of the disulfide relay system substrate COX17, perhaps as a compensatory effect. Interestingly, three patients with a mutation in GFER were identified to have a progressive mitochondrial myopathy that presented with decreased activity of complex IV (Di Fonzo et al., 2009). Electron micrographs of their skeletal muscle revealed enlarged mitochondria, and the appearance of vacuole-like structures (Di Fonzo et al., 2009), similar to our observations in *Grx2^−/−^* muscle. These findings indicate that the disulfide relay system may be linked to the activation of mitophagy, as both *Grx2* deletion and GFER mutation resulted in abnormal mitochondrial ultrastructure and the appearance of vacuole-like structures (Di Fonzo et al., 2009). This emerging mechanistic link between GFER and autophagy is supported by *in vitro* evidence demonstrating that genetic depletion of GFER is associated with the activation of autophagy (Todd et al., 2010; Pu et al., 2017), in part through the AMPK/mTOR pathway (Pu et al., 2017).

Our findings demonstrate that *Grx2* is involved in the regulation of mitochondrial morphology, autophagy, and energetics in skeletal muscle (Figure 8). The highly irregular ultrastructure of mitochondria and appearance of multi-lamellar structure in TEM micrographs of *Grx2^−/−^* muscle, combined with the increase in protein expression of parkin, elevated LC3II/I flux, and the colocalization of mitochondria and lysosomes in Grx2 deficient primary myoblasts are collectively consistent with the conclusion that there is increased autophagic clearance of mitochondria in skeletal muscle of *Grx2^−/−^* mice. Deletion of *Grx2* decreased the GSH:GSSG ratio, and this was associated with increased mitochondrial length, which also suggests that the absence of *Grx2* promotes stress-induced mitochondrial hyperfusion. These structural abnormalities in mitochondria were associated with impaired complex I OXPHOS activity in myofibers in *Grx2* deficient muscle. Future studies should further examine the molecular mechanisms that contribute to the glutathione redox control of autophagy. Moreover, studies should investigate the role of glutathione redox and *Grx2* in mammalian diseases associated with impaired complex I and IV function and assembly.

## Data Availability Statement

The raw data supporting the conclusions of this article will be made available by the authors, without undue reservation.

## Ethics Statement

The animal study was reviewed and approved by Animal Care Committee of the University of Ottawa.

## Author Contributions

Conceived and designed the experiments: M-EH. Performed the experiments: AL, CP, GP, DP, NH, AC. Analyzed data: CP, AL, GP, DP, NH. Drafted the manuscript: CP, GP. Critically evaluated and contributed to the manuscript: CP, GP, DP, NH, AC, YB, M-EH. All authors contributed to the article and approved the submitted version.

### Conflict of Interest

The authors declare that the research was conducted in the absence of any commercial or financial relationships that could be construed as a potential conflict of interest.

## Footnotes

^1^Raw microarray data are available at http://www.genenetwork.org.

## Abbreviations

GRX2: Glutaredoxin 2
DRP1: Dynamin related protein-1
GFER: Growth factor, augmenter of liver regeneration
GSH: Reduced glutathione
GSSG: Oxidized glutathione disulfide
IMS: Mitochondrial intermembrane space
MFN: Mitofusin
MFF: Mitochondrial fission factor
Mia40: Mitochondrial intermembrane space import and assembly protein 40
OPA1: Optic atrophy type 1
OXPHOS: Oxidative phosphorylation
RER: Respiratory exchange ratio
ROS: Reactive oxygen species
TEM: Transmission electron microscopy
UCP3: Uncoupling protein 3.
